# Bridging the gender gap in eye health by training allied ophthalmic personnel

**Published:** 2025-03-07

**Authors:** Rutul Shah, Shrikant Ayyangar, Franklin Daniel, Elizabeth Kurian

**Affiliations:** 1 Manager –Training and Quality, Allied Ophthalmic Personnel (AOP) Training Programme, Mission for Vision, Mumbai, India.; 2 Lead – Communications, Mission for Vision, Mumbai, India; 3 Lead – Vision Centre & Allied Ophthalmic Personnel (AOP) Training Programme, Mission for Vision, Hyderabad, India.; 4 Chief Executive Officer, Mission for Vision, Mumbai, India.

**Young women trained as eye care professionals are serving as role models and increasing the likelihood that other women will seek eye care.**

**Figure:**
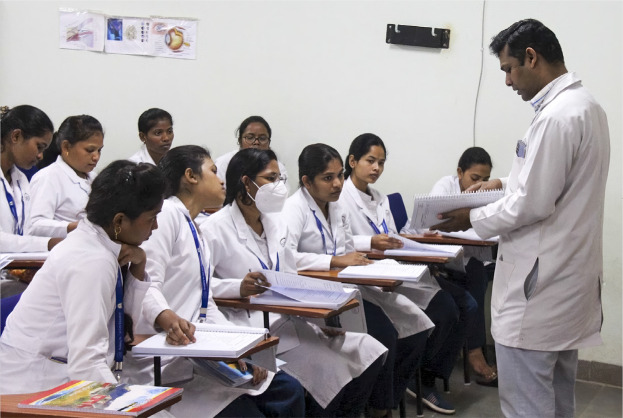
Women from disadvantaged backgrounds are trained to become allied ophthalmic personnel. india

© MISSION FOR VISION CC BY-NC-SA 4.0

In India, women, especially from marginalised communities, are disproportionately affected by blindness. They are 1.4 times more likely to be affected by visual impairment than men.^1^ Traditional gender roles that prioritise men's health, financial limitations, and a lack of female eye care professionals remain significant challenges for women.

The Mission Saksham initiative in India was established to train young women from disadvantaged backgrounds who have completed Grade 12 to become allied ophthalmic personnel (AOPs) in the eye care sector. India requires over 98,000 AOPs to meet basic eye care needs and achieve good universal eye health coverage.^2^ Mission Saksham addresses this shortage by training AOPs to provide essential eye care services and take on leadership roles within their communities. As of 2024, 92% of participants are women, with over 500 AOPs trained to lead vision centres, manage clinics, and assist during surgery.

The female AOPs act as role models and change agents, driving equity and better health outcomes in their communities. For example, two women from the first cohort of trainees, who completed their training at the LV Prasad Eye Institute (LVPEI) in 2017, are now managing a vision centre in their hometown in the state of Meghalaya. They are not only providing essential eye care but also leading their community's eye care services. One of them, Marbalin Wanniang, said: “On average, I attend to between 10 and 25 patients on market days, with 60% of these being women and girls. Female patients tend to feel more comfortable with a female eye health professional, even if they haven't explicitly mentioned it.”

Research has shown that women in leadership roles in health care not only improve access to services but also enhance the quality of care, particularly for women and children.^1^

**Saniya's Journey**

**Figure:**
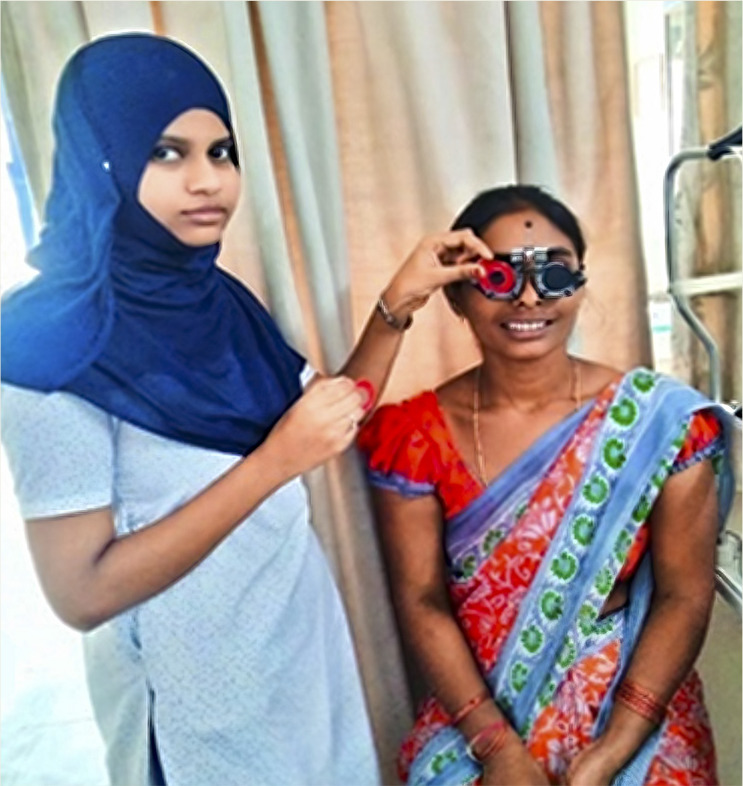
Saniya conducting a refraction test at the vision centre in Kothakota, Telangana. india

© MISSION FOR VISION CC BY-NC-SA 4.0

Saniya grew up in a single-parent household, with her mother working as a tailor to support her and her brother. After completing the Mission Saksham allied ophthalmic personnel course at the LV Prasad Eye Institute, Saniya was appointed as a vision technician at the vision centre in Kothakota, in the state of Telangana. Between January and September 2024, Saniya examined 2,328 patients, 48% of whom were women. She dispensed spectacles to 483 patients, achieving a 75% conversion rate, with 55% being women.

Of the 258 patients she referred, 212 underwent surgery; 53% were women. Many patients, especially elderly women from marginalised backgrounds, were reluctant to see male professionals. Since Saniya started working at the vision centre, the women began to seek treatment for themselves and to refer other women. Saniya says: “Before I became a vision technician, many women in my community were reluctant to visit male eye care professionals. Now, they trust me to help them with their eye health.”
